# Mimivirus-like Particles in Acanthamoebae from Sewage Sludge

**DOI:** 10.3201/eid1706.101282

**Published:** 2011-06

**Authors:** William H. Gaze, Gina Morgan, Lihong Zhang, Elizabeth M.H. Wellington

**Affiliations:** Author affiliation: University of Warwick, Coventry, UK

**Keywords:** Mimiviridae, sewage, disease reservoirs, refuse disposal, communicable diseases, emerging, Acanthamoeba, viruses, letter

**To the Editor:** Mimivirus is a giant, double-stranded DNA virus. Its 650-nm diameter and 1.2-Mb genome make it the largest known virus (*1*). In 2003, mimivirus was isolated from a water cooling tower in Bradford, UK, after a pneumonia outbreak and was reported to infect *Acanthamoeba polyphaga* amebae (*2*). Subsequently, a small number of additional isolates have been reported (*3*).

Mimivirus has been associated with pneumonia, and this association was strengthened after antibodies to mimivirus were found in serum samples from patients with community- and hospital-acquired pneumonia and after mimivirus DNA was found in bronchoalveolar lavage specimens (*4*). More direct evidence of pathogenicity was illustrated when a pneumonia-like disease developed in a laboratory technician who worked with mimivirus and showed seroconversion to 23 mimivirus-specific proteins (*5*).

We report finding mimivirus-like particles during our molecular study of *Acanthamoeba* spp. abundance and diversity in final-stage conventionally treated sewage sludge from a wastewater treatment plant in the West Midlands, UK. Using metagenomic DNA extracted from the sludge (*6*), we estimated the abundance of *Acanthamoeba* spp. by using real-time PCR (*7*) and found it to be ≈1 × 10^2^/g sludge. To assess species diversity, we amplified an *Acanthamoeba* spp.–specific 18S rRNA target, which resulted in products of ≈450 bp (*8*). PCR products were cloned and sequenced, revealing low *Acanthamoeba* spp. diversity with a predominance of clones most similar to *A. palestinensis* (22/25 clones), which fall within the T6 clade according to the classification of Stothard et al. (*9*). A small number (3/25) of clones showed closest similarity to acanthamoebae belonging to the T4 clade, which includes strains considered to be human pathogens, including some *A. polyphaga* strains.

Acanthamoebae were isolated from fully digested sewage sludge by inoculating diluted sludge onto cerophyl-Prescott infusion agar and subculturing onto nonnutrient agar plates streaked with heat-killed *Escherichia coli*. Cultures were incubated at 20°C and 30°C and examined under an Axioskop 2 microscope (Zeiss, Oberkochen, Germany) at 100× magnification; cells of interest were examined at 1,000× magnification. One clonal population of an *Acanthamoeba* sp. isolated at 20°C, which demonstrated typical trophozoite and cyst morphology, contained large numbers of particles either within vacuoles or within the cytoplasm. Vacuoles were densely packed with particles that appeared to be constantly moving; vacuole size varied from that typical of food vacuoles to large vacuoles that occupied most of the cell volume (Figure, panels B, D, and G). Because the particles were assumed to be bacterial pathogens, efforts were made to produce an axenic culture of the ameba isolate, and 16S rRNA PCR was performed to identify any intracellular bacteria. DNA was extracted by using a phenol chloroform method according to Griffiths et al. (*6*). However, no 16S rRNA PCR products were amplified.

**Figure:**
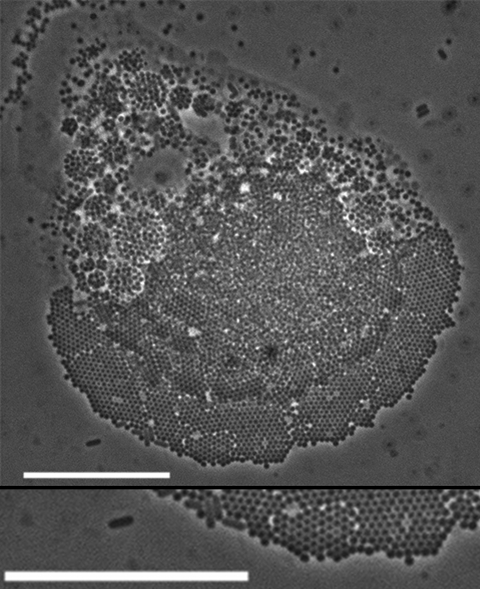
Light micrograph images of acanthamoebae infected with mimivirus-like particles, showing cells packed with mimivirus-like particles. Enlargement of region of image is shown at bottom of panel A. White arrows in panels B and D indicate free virus-like particles. pVF, putative viral factory. Scale bars = 10 µm.

Months later, an image review led to recognition of unusual arrangements of intracellular particles in a lattice-like structure in which each particle was surrounded by 6 others. Measurement of rows of particles, assuming tight packing, gave an average particle size of 620 nm. At this point, we realized that the particles were virus-like and closely resembled mimivirus. Subsequent efforts to resuscitate the infected *Acanthamoeba* spp. culture were unsuccessful, and performing specific PCR for mimivirus sequences was not possible. Sewage sludge samples collected later were tested for mimivirus by using PCR (*4*); however, no amplification was observed, indicating either that mimivirus was present only transitorily, that mimivirus was below detection limits, or that the target primer sites were not conserved.

The density of virus-like particles within acanthamoebae cells was extremely high (Figure, panel A). The advantage of in situ observation of amebae on the surface on which they were cultivated is that the cell is not disturbed. The virus-like particles are arranged in tightly packed, flat sheets, indicative of an icosahedral structure. Toward the bottom of Figure panel A, a single sheet of particles can be seen, corresponding to the hyaline zone at the anterior of the cell, and in the center of the cell are multiple layers of tightly packed virus-like particles. Toward the anterior of the cell, vacuoles containing particles were apparently being egested at the uroid. Dense 3-dimensional aggregations of particles (Figure, panel E) resembled previously described virus factories (*10*). Free mimivirus-like particles (Figure, panels B, D) indicate egestion by amebae or the result of amebal lysis, a phenomenon observed in cocultures. Prevalence of infection was high and infection was immediately obvious, even when cultures were observed at low magnification (100×).

Although we did not confirm the identity of the mimivirus-like particles by molecular methods or electron microscopy, the nature of the light micrographs enabled close examination of the particles. These particles demonstrated close similarity to mimivirus in size and shape as indicated by the lattice arrangement in which 1 particle was surrounded by 6 others, as seen previously (*10*). Our study illustrates that acanthamoebae that survive sewage treatment can harbor mimivirus-like particles, which could be disseminated to agricultural land and surface waters.
